# Tethered Cation Size Affects the Imbibition of Polymerized Ionic Liquids and the Ionic Conductivity in Nanopores

**DOI:** 10.1021/acs.macromol.5c01449

**Published:** 2025-07-07

**Authors:** Yun Dong, Hongkun He, Kriti Kapil, Martin Steinhart, Krzysztof Matyjaszewski, Hans-Jürgen Butt, George Floudas

**Affiliations:** † Max Planck Institute for Polymer Research, 55128 Mainz, Germany; ‡ Department of Chemistry, 6612 Carnegie Mellon University, Pittsburgh, Pennsylvania 15213, United States; § Institut für Chemie neuer Materialien, 9186 Universität Osnabrück, D-49069 Osnabrück, Germany; ∥ Department of Physics, University of Ioannina, 45110 Ioannina, Greece; ⊥ University Research Center of Ioannina (URCI) - Institute of Materials Science and Computing, 45110 Ioannina, Greece

## Abstract

There is a growing interest in new polymerized ionic liquids (PILs) with enhanced ion transport properties especially at low temperatures, in the vicinity of the liquid-to-glass temperature, *T*_g_. We employed two structurally similar ionic liquids (ILs), namely, 1-butyl-3-methylimidazolium bis­(trifluoromethylsulfonyl)­imide ([BMIM]^+^[TFSI]^−^) and 1-(4-vinylbenzyl)-3-butylimidazolium bis­(trifluoromethane)­sulfonimide ([VBBI]^+^[TFSI]^−^) and synthesized the corresponding PILs, poly­[BVIM]^+^[TFSI]^−^, and poly­[VBBI]^+^[TFSI]^−^. The main difference was the positioning of the cation—and hence the cation/anion coordination—with respect to the backbone. This small structural variation had implications in anion transport in the bulk. Ion coordination in proximity to the backbone restricted backbone mobility, increased *T*_g_, and reduced ionic conductivity. A strategy toward increasing ion conductivity at lower temperatures was by nanometer confinement. We employed as a confining medium self-ordered anodic aluminum oxide (AAO) nanopore templates and investigated the kinetics of imbibition and the ion dynamics following imbibition by *ex situ* polarizing optical microscopy and by *in situ* nanodielectric spectroscopy. These methods provided access to the effective viscosity and to the ionic conductivity of a PIL during and following imbibition in nanopores. PILs penetrated nanopores with a lower speed than expected from their bulk viscosity. At lower temperatures, in the vicinity of *T*_g_, confinement effects took over and decoupled the ion dynamics from the arrested backbone dynamics. Under these conditions, the temperature dependence of ion conductivity deviated from the Vogel–Fulcher–Tammann law and followed an Arrhenius temperature dependence with an activation energy that was reduced from 142 kJ/mol in the bulk to ∼108 kJ/mol under confinement. The results offer new insights into how molecular structure and confinement affect ion transport in PILs.

## Graphical abstract

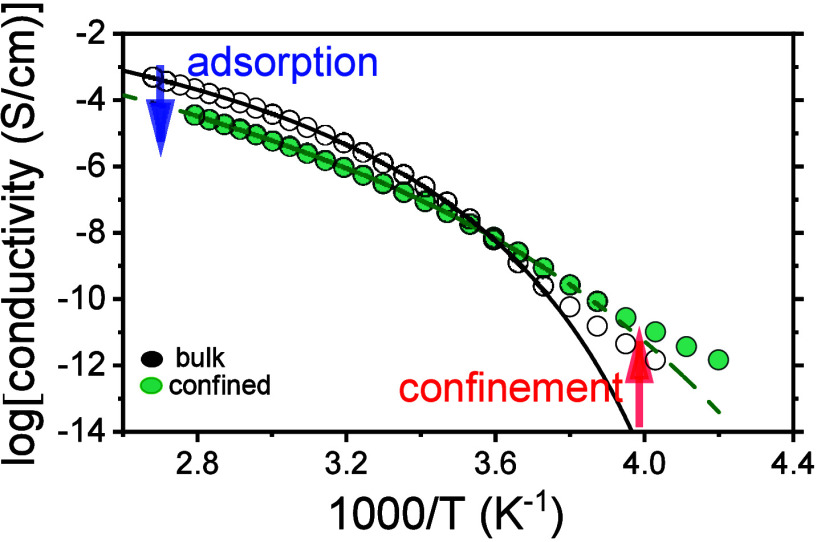

## Introduction

Polymerized ionic liquids (PILs) are a unique class of materials composed of polymer backbones bearing covalently attached ionic groups.^1,2^ Unlike conventional ionic liquids (ILs), i.e., low-molecular-weight salts composed solely of mobile ions, PILs incorporate at least one ionic species–typically the cation–into the polymeric chain, while the counterion remains mobile.^3,4^ This hybrid architecture combines the desirable physicochemical features of ILs (e.g., high ionic conductivity,^5,6^ tunability, and thermal stability^7^) with the mechanical robustness and processability of polymers.^8^ The cationic components in PILs are often based on imidazolium, pyridinium, ammonium, or phosphonium structures, while the anions include bis­(trifluoromethylsulfonyl)­imide ([TFSI]^−^), tetrafluoroborate ([BF_4_]^−^), or hexafluorophosphate ([PF_6_]^−^).^9–11^ The chemical diversity of these ions allows for fine-tuning of the properties to meet specific requirements in a range of electrochemical and soft material applications. These include solid-state electrolytes for lithium-ion batteries and supercapacitors, ion- conducting membranes for fuel cells and electrodialysis, actuators, sensors, and stimuli-responsive materials.^12–17^ Their high ionic content, combined with low volatility, nonflammability and thermal stability, makes them particularly attractive for replacing liquid electrolytes in next-generation energy storage and conversion technologies.

In recent years there has been a growing interest in employing PILs within nanostructured environments, such as nanoporous materials, in an effort to modulate or even to enhance their ionic transport properties. Under confinement, the ion dynamics can be altered because of spatial constraints and surface interactions leading to adsorption.^18–21^ In the first study^18^ of the archetypal polymer electrolyte poly­(ethylene oxide)/LiTFSI during imbibition in nanopores by *in situ* nanodielectric spectroscopy, it was shown that ion conductivity is controlled by PEO adsorption at the pore walls. The molar mass dependence of the characteristic adsorption times (τ_ads_ ∼ *N* ^2^, *N* is the degree of polymerization) was in agreement with the scaling theory proposed by de Gennes. In another study of PIL/IL mixtures, it was shown that *in situ* conductivity measurements during imbibition in nanopores can used to separate the mixture to its individual components.^19^ However, despite the growing body of research on ILs and PILs,^22–27^ relatively little is known about how subtle molecular modifications, such as side-group variations in PILs, influence their behavior in bulk and under confinement. Especially, the effect of polymer adsorption and the concomitant immobilization of the cation on the pore walls on the (anion) mobility needs to be investigated. Molecular architecture/topology as well as confinement effects can influence cation–anion association, which is expected to affect ion conductivity.

In this work, we focus on how the cation molecular structure affects ion transport in ILs and the corresponding PILs in the bulk and under nanometer confinement. For this purpose, two structurally similar ILs were synthesized: one bearing a simple imidazolium ring (1-butyl-3-methylimidazolium, [BMIM]^+^), the other featuring a phenyl-substituted imidazolium (1-(4-vinylbenzyl)-3-butylimidazolium, [VBBI]^+^), while keeping the same anion, bis­(trifluoromethylsulfonyl)­imide [TFSI]^−^. Next, the respective polymers were synthesized comprising: poly­[BVIM]^+^[TFSI]^−^ and poly­[VBBI]^+^[TFSI]^−^. The results show that the more compact cation structure in the former allows for anion/cation association more closely to the polymer backbone, effectively restricting backbone mobility. The expectation is that this feature will reduce ion conductivity. Building on this molecular-level understanding of ion transport, we focus particularly on the role of cation structure in modulating ion dynamics during and following flow of PILs in nanopores. For this purpose, we employ self-ordered anodic aluminum oxide (AAO) nanopores, and a combination of dielectric spectroscopy, thermodynamic and viscosity measurements, to probe how confinement, polymer-AAO interactions, and ion-polymer interactions collectively influence ion conductivity and ion relaxation. Of particular interest is the possibility to decouple the ion motion from the polymer segmental dynamics, known as ion-polymer decoupling.^28–31^ According to the latter, ion motion can decouple from the polymer backbone dynamics and persist even below the liquid-to-glass temperature (*T*_g_). We investigate how this decoupling is impacted by cation structure in the bulk and under spatial confinement. With respect to the latter, we show that confinement enhances ion decoupling below *T*_g_. The activation energy for ion diffusion reduces from 142 kJ/mol in the bulk to 108 kJ/mol under confinement. The results offer new insights into how molecular structure (cation size in particular) and confinement effects affect ion transport in PILs.

## Experimental Section

### Materials

1-Butyl-3-methylimidazolium bis­(trifluoromethylsulfonyl)­imide ([BMIM]^+^[TFSI]^−^, purity ≥ 98%), 1-butyl-3-vinylimidazolium bromide ([BVIM]^+^[Br]^−^, purity ≥ 98%), 4-vinylbenzyl chloride (purity ≥ 90%), 1-butylimidazole (purity ≥ 98%), Lithium bis­(trifluoromethanesulfonyl)­imide (LiTFSI, purity ≥ 98%), Acetonitrile (purity ≥ 99.8%), Dichloromethane (DMF, extra dry ≥ 99.9%), Silver nitrate solution (AgNO_3_), 2,2’-azobis­(isobutyronitrile) (AIBN, purity ≥ 98%), Ultra-15 Centrifugal Filter Unit (10 kDa MWCO), tetrahydrofuran (THF, purity ≥ 98%), anhydrous MgSO_4_, and ethanol were purchased from Merck and used without further purification. Deionized water was purified using a Millipore Milli-Q Synergy with a resistivity approximately 18 MΩ·cm^–1^. The water content of ILs was measured by a Karl Fischer titration method (C20, Mettler Toledo). Following freeze-drying the water content was below 0.02 wt %. The chemical structures of the ILs and PILs used in this study are presented in Scheme 1.

**Scheme 1:**
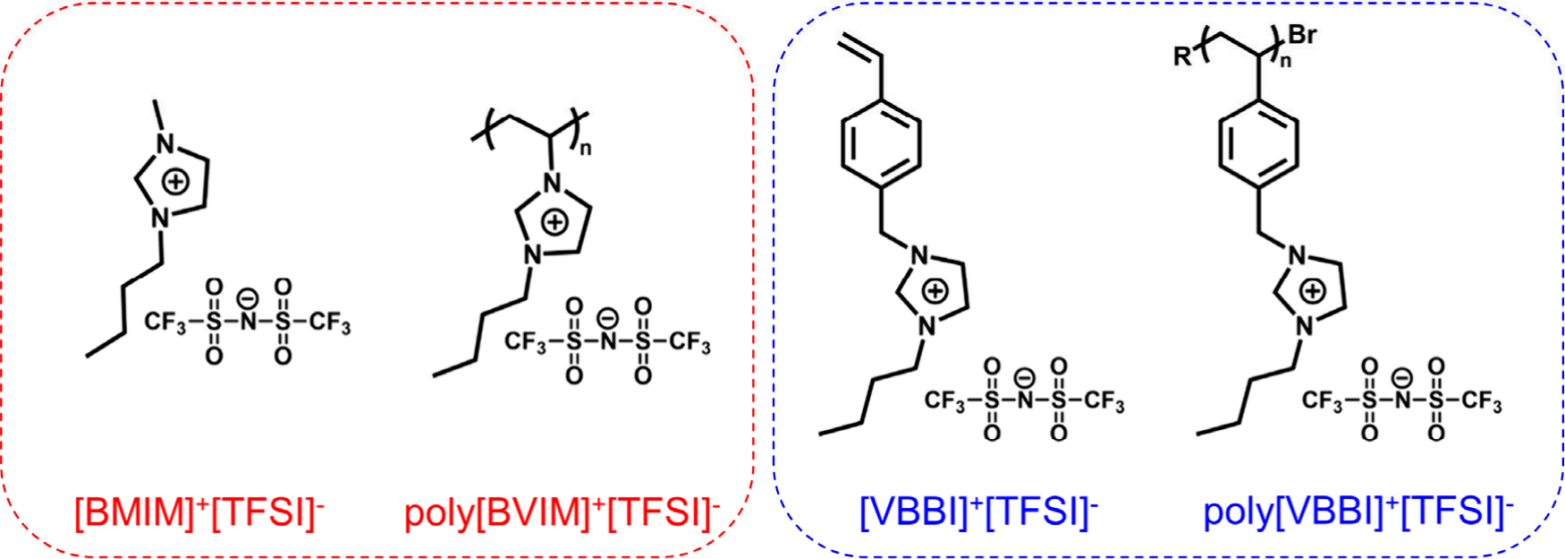
Chemical Structures of ILs, [BMIM]^+^[TFSI]^−^, [VBBI]^+^[TFSI]^−^, and of the Corresponding PILs, poly­[BVIM]^+^[TFSI]^−^, poly­[VBBI]^+^[TFSI]^−^

### Synthesis of [VBBI]^+^[TFSI]^−^

The preparation of IL, [VBBI]^+^[TFSI]^−^, was conducted according to an established procedure.^32^ 4-vinylbenzyl chloride (40.0 g, 0.26 mol) was mixed with 1-butylimidazole (32.5 g, 0.26 mol) in acetonitrile (100 mL), and the reaction mixture was stirred at 45 °C for 24 h. The resulting product, 1-(4-vinylbenzyl)-3-butylimidazolium chloride (VBIC), was precipitated, washed thoroughly with excess diethyl ether, and dried under vacuum at room temperature to yield a viscous liquid. Subsequently, VBIC (27.0 g, 96.1 mmol) was dissolved in deionized water (200 mL), and LiTFSI (30.4 g, 105.8 mmol) was added. An oily liquid immediately formed at the bottom of the reaction flask. The mixture was stirred at room temperature for 24 h, after which the oil was extracted with ethyl acetate (200 mL) and washed with deionized water (3 × 50 mL). The organic phase was dried over anhydrous MgSO_4_, filtered, and concentrated by rotary evaporation. Residual solvent was removed under a vacuum, yielding a highly viscous liquid identified as 1-(4-vinylbenzyl)-3-butylimidazolium bis­(trifluoromethane)­sulfonimide ([VBBI]^+^[TFSI]^−^). The corresponding PIL, poly­[VBBI]^+^[TFSI]^−^, was synthesized using atom transfer radical polymerization (ATRP).^33–37^ ATRP is a controlled/living radical polymerization and is used to prepare polymers with high uniformity and well-defined architecture.

### Synthesis of Poly­[BVIM]^+^[TFSI]^−^

The synthesis of poly­[BVIM]^+^[TFSI]^−^ was made following a previously reported procedure.^38,39^ Briefly, [BVIM]^+^[Br]^−^ (9 g, 38.9 mmol) and AIBN (6.5 mg, 0.396 mmol) were dissolved in 18 mL of DMF. The solution was purged with argon for 1 h to remove dissolved oxygen. The reaction mixture was then stirred at 348 K under an argon atmosphere for 24 h. Following the reaction, DMF was removed via rotary evaporation, yielding a viscous oil. This crude product was dissolved in 20 mL of deionized water and dialyzed against water using a cellulose tube with 10 kDa MWCO for 2 days, with the dialysate being replaced twice to ensure effective removal of low-molecular-weight impurities. To facilitate anion exchange, the dialyzed solution was divided into three equal portions. Each portion was treated with an aqueous solution of LiTFSI (approximately 1.5-fold molar excess) and stirred for 5 days. The resulting polymer was isolated by filtration, thoroughly washed with deionized water until no bromide could be detected in the washing water (confirmed by the absence of AgBr precipitation upon the addition of AgNO_3_ solution). Finally, the obtained poly­[BVIM]^+^[TFSI]^−^ (4.48 g, *M*_n_ = 85 kDa) was dried under vacuum at 313 K to remove residual solvent. Gel permeation chromatography (GPC) was used to determine the molecular weights of obtained PILs (Figure S1). The molecular characteristics of poly­[BVIM]^+^[TFSI]^−^ and poly­[VBBI]^+^[TFSI]^−^ are given in Table 1.

**Table 1:** Molecular Characteristics of PILs (Number- and Weight-Averaged Molar Mass, Degree of Polymerization and Dispersity) Used in the Present Investigation

	*M*_*n*_ (kg·mol^–1^)	*M*_*w*_ (kg·mol^–1^)	DP	*Đ*
poly[BVIM]^+^[TFSI]^−^	85	255	525	3.02
poly[VBBI]^+^[TFSI]^−^	180	246	477	1.36

### AAO Templates

Self-ordered nanopores aluminum oxide (AAO) templates with specified pore diameters of 25, 35, 65, and 400 nm, and a uniform pore depth of approximately 100 μm, were fabricated following established literature protocols.^40–43^ The AAO layers were supported on 900 μm thick aluminum substrates. Prior to the infiltration process, all AAO templates were annealed in a vacuum oven at 433 K for 12 h. This annealing step eliminates a substantial portion of hydroxyl (−OH) groups from the surface of the AAO. The templates have been characterized by different methods including SEM, FIB, AFM^44^ and laser interferometry.^45^ The optical experiments have shown that AAO nanopores are characterized by a slight conicity. A model provided an improved description of nanoscale fluid dynamics and allowed geometric characterization of the nanoporous membranes by their imbibition kinetics.^45^

### Differential Scanning Calorimetry (DSC)

The thermal behavior of ILs and their corresponding PILs was investigated using differential scanning calorimetry (DSC) during cooling and subsequent heating cycles at a rate of 10 K·min^–1^. Measurements were performed with a Mettler Toledo DSC-822 calorimeter, using an empty aluminum pan as a reference. Samples were accurately weighed with a Mettler Toledo AX205 balance, and approximately 10 mg of each sample was sealed in 100 μL aluminum pans. To eliminate prior thermal history, samples were heated to 373 K under a nitrogen atmosphere before further analysis. Each thermal cycle was repeated twice to ensure reproducibility.

### Dielectric Spectroscopy (DS)

The dielectric properties of bulk and confined ILs, PILs were performed by usual dielectric spectroscopy and *in situ* nanodielectric spectroscopy (*n*DS). In both cases, a Novocontrol Alpha frequency analyzer was used, which consists of a broadband dielectric converter and an active sample head. For bulk samples, a dielectric cell with two electrodes (20 mm diameter) and a 250 μm Teflon spacer to ensure constant thickness was used. The frequency range employed spanned from 10^–2^ to 10^7^ Hz. For the investigation of the effect of nanoconfinement, a 35 nm thick gold layer (9 mm diameter) was deposited onto the AAO template to serve as the top electrode, while the aluminum substrate of the AAO acted as the bottom electrode. Deposition of a gold layer was carried out via sputtering under vacuum conditions (better than 2 × 10^–5^ Pa) using a Bal-tec MED 020 system with a current density of 35 mA. The samples were then placed on top of the AAO template, and imbibition kinetics were investigated using *n*DS. In this case, a narrower frequency range (1–10^6^ Hz) was employed for faster data acquisition.

Impedance measurements were conducted to obtain the complex conductivity function, denoted as σ* = σ′ + iσ″, where σ′ and σ″ are the real (*i.e*., the dc-conductivity) and imaginary parts, respectively. The plateau observed in the real part σ′ was used to extract the dc-conductivity. *n*DS can be used to monitor the increase in imbibition length during pore filling by tracking the rise in dc-conductivity with imbibition time. Briefly, a simple Debye model for the frequency-dependent complex conductivity (σ*­(ω)) can be employed as σ*­(ω) = σ′(ω) + *iσ*″(ω) = *iωε*_0_ ε*­(ω),^46,47^

$$\sigma^{\prime }\left(\omega \right)=\omega \varepsilon_{0}\varepsilon^{\prime \prime }\left(\omega \right)=\sigma_{dc}+\frac{\omega^{2}\tau \varepsilon_{0}\Delta \varepsilon }{1+{\left(\omega \tau_{D}\right)}^{2}}$$ 1

$$\sigma^{\prime \prime }\left(\omega \right)=\omega \varepsilon_{0}\varepsilon^{\prime }\left(\omega \right)=\omega \varepsilon_{0}\left(\varepsilon_{\infty }+\frac{\Delta \varepsilon }{1+{\left(\omega \tau_{D}\right)}^{2}}\right)$$ 2

Here, *Δε* is the dielectric strength, τ_*D*_ is the characteristic relaxation time, σ_dc_ is the dc-conductivity, and *ε*_∞_ is the dielectric permittivity in the limit of very high frequencies. A parallel model can describe the total equivalent capacitance, comprising the capacitance of the sample (*C*_1_) and air (*C*_2_) connected in series, whereas the combined sample/air capacitance (*C*_12_) connects in parallel with the AAO (*C*_3_). As a result,

$$\frac{1}{C_{12}}=\frac{1}{C_{1}}+\frac{1}{C_{2}},\;and\;C_{123}=C_{12}+C_{3}$$ 3

Using the above equations, the definition σ_123_^*^ = *iωε*_0_ ε*, and the porosity φ_12_ results in

$$\sigma_{123}^{*}=\sigma^{\prime }+i\sigma^{\prime \prime }=\left\{\left[\frac{Ld_{1}\sigma_{1}^{\prime }{\left(\omega \varepsilon_{0}\right)}^{2}}{{\left(d_{2}\sigma_{1}^{\prime }\right)}^{2}+{\left(d_{1}\omega \varepsilon_{0}+d_{2}\sigma_{1}^{\prime \prime }\right)}^{2}}\right]\varphi_{12}+\sigma_{3}^{\prime }\varphi_{3}\right\}+i\left\{\left[\frac{L\left[d_{2}\left[{\left(\sigma_{1}^{\prime }\right)}^{2}+{\left(\sigma_{1}^{\prime \prime }\right)}^{2}\right]\omega \varepsilon_{0}+\sigma_{1}^{\prime \prime }{\left(\omega \varepsilon_{0}\right)}^{2}d_{1}\right]}{{\left(d_{2}\sigma_{1}^{\prime }\right)}^{2}+{\left(d_{1}\omega \varepsilon_{0}+d_{2}\sigma_{1}^{\prime \prime }\right)}^{2}}\right]\varphi_{12}+\sigma_{3}^{\prime \prime }\varphi_{3}\right\}$$ 4

where φ_3_ (= 1 – φ_12_) is the AAO content. As have been reported previously,^48^ eq 4 predicts a conductivity plateau (e.g., the dc-conductivity), only when the pores are fully infiltrated.

### X-ray Diffraction

X-ray diffraction measurements were performed using a Rigaku SmartLab X-ray diffractometer. The X-ray source was a rotating Cu anode, operating at 45 kV and 200 mA. The incident optics included a Ge crystal monochromator (220 reflection) followed by a parallel slit (incident Soller slit 2.5°). A diffracted beam monochromator was inserted between the detector slit and the detector to minimize fluorescence and K_β_ radiation. The receiving optics consisted of a parallel slit analyzer (PSA) followed by a receiving Soller slit. The X-ray wavelength was 0.154059 nm. Data were collected using a HyPix-3000 2D detector (pixel size: 100 μm × 100 μm; active area: 38.5 mm × 77.5 mm). The scans were conducted in the 2Θ range from 0° to 60° with a step size of 0.01° at 358 K.

### Reflection Optical Microscopy

The imbibition length measurements were made by employing *ex situ* reflection optical microscopy (ROM) (Zeiss Axiotech vario) at 358 K (infiltration temperature). The PIL, poly­[VBBI]^+^[TFSI]^−^, was infiltrated into nanopores by capillary force. At predefined time intervals, the AAO templates were immersed into liquid nitrogen to prevent further infiltration. The imbibition length, representing the distance the PIL penetrated under capillary pressure, was determined from cross-sectional images captured by ROM.

### Contact Angle

The apparent advancing contact angle of poly­[VBBI]^+^[TFSI]^−^ was measured by placing nearly spherical polymer particles (∼1 mm in diameter) onto an electropolished Al substrate. To form the spherical particle, around 1 mg of sample was placed on a superamphiphobic surface, heated to 393 K, and maintained under a vacuum for 24 h to allow surface tension to shape the polymer into spheres. The polymer spheres were then slowly cooled and transferred onto the electropolished Al disk, which was coated with a thin native oxide layer to simulate the AAO surface. The contact angle was measured using a commercial goniometer (OCA35, DataPhysics) with IDS uEye software, at 358 K which corresponds to the infiltration temperature.

### Surface Tension

The Wilhelmy plate method was employed to measure the surface tension of poly­[VBBI]^+^[TFSI]^−^. A platinum–iridium rod with a diameter of 1.2 mm was vertically immersed into the heated PIL (at 358 K). The downward force (*F*) exerted by the surface tension was measured using a tension meter (DCAT 11BC, DataPhysics) after allowing the system to equilibrate for approximately 2000 s. The surface tension (γ) was then calculated from *F* = *lγ*, where *l* is the circumference of the rod.

### Rheology

The zero-shear viscosity was investigated using a shear rheometer (ARES) equipped with an environmental test chamber. Measurements were conducted as a function of temperature. Samples were placed on the lower plate of an 8 mm diameter parallel plate geometry, with the upper plate brought into contact and the sample thickness adjusted. The storage (*G′*) and loss (*G″*) shear moduli were monitored as a function of frequency, ω, in the range 10^–1^ < ω < 10^2^ rad·s^–1^, within the linear viscoelastic regime. The complex viscosity (η*) was calculated using η* = *G″*/ω – *iG′*/ω. Master curves were made by use of the time–temperature superposition principle (*tT*_s_). The latter allows the frequency ω dependence of the complex modulus G* at any temperature *T* to be determined from a master curve at a reference temperature. At each temperature *T*, a single frequency-scale shift factor (*a*_*T*_) enables the superposition of all viscoelastic data at temperature *T* onto the data at the reference temperature, *T*_ref_, as G*(*ω*;*T*) = G*­(a_T_ *ω*;*T*_ref_). Master-curves used to extract the zero-shear viscosity, η_0_, are presented in Figures S2.

## Results and Discussion

### Bulk Properties

The thermodynamic properties of two ILs sharing the same anion, [TFSI]-, and of the corresponding PILs are strongly influenced by cation molecular structure and size. Figure 1 presents the DSC traces, highlighting these differences. The cooling traces (rate of 10 K·min^–1^) reveal a step-like decrease of heat flow at the glass temperature (*T*_g_), representing the transformation from the liquid to the glass. All samples were found to be fully amorphous, with the exception of [BMIM]^+^[TFSI]^−^ that exhibited cold-crystallization and melting during heating. Among the ILs, [VBBI]^+^[TFSI]^−^ exhibits a higher *T*_g_ compared to [BMIM]^+^[TFSI]^−^. It can be attributed to the reduced mobility introduced by the extra phenyl group within [VBBI]^+^. However, in the PILs, it is the poly­[BVIM]^+^[TFSI]^−^ that exhibits the higher *T*_g_. The proximity of the imidazolium cation to the main chain in poly­[BVIM]^+^[TFSI]^−^ restricts the backbone mobility and gives rise to a higher *T*_g_ as compared to poly­[VBBI]^+^[TFSI]^−^.

**Figure 1:**
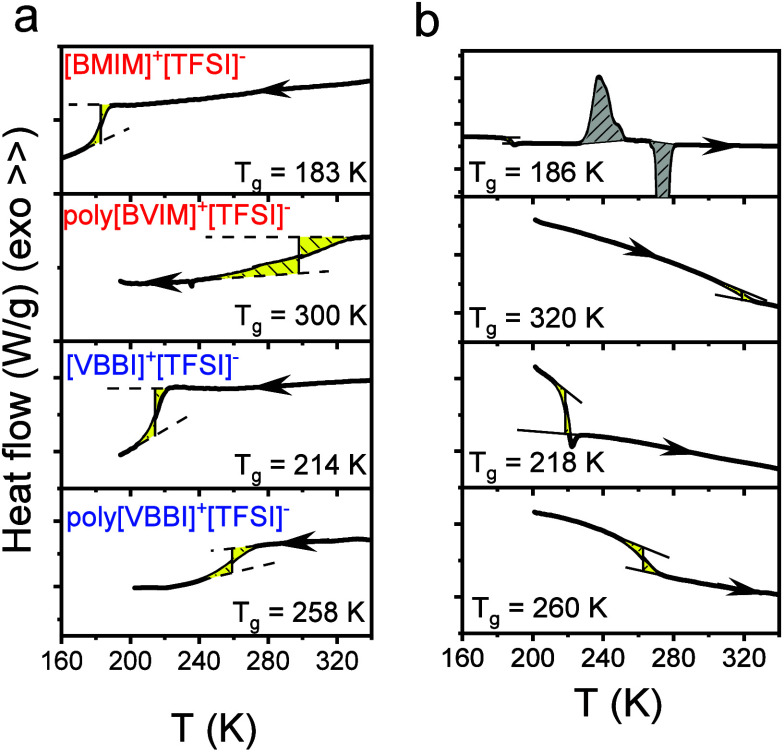
DSC traces of ILs, [BMIM]^+^[TFSI]^−^, [VBBI]^+^[TFSI]^−^, and the corresponding PILs, poly­[BVIM]^+^[TFSI]^−^, and poly­[VBBI]^+^[TFSI]^−^ in the bulk. Traces are shown during cooling (a) and subsequent heating (b) runs with a rate of 10 K·min^–1^.

ILs (and PILs) have a higher level of organization as compared to normal liquids.^19,48^ Information about their local packing can be obtained through X-ray diffraction. The diffraction patterns of the ILs and their corresponding PILs are shown in Figure 2a. In the ILs, arrows 1, 2, and 3 correspond to the smectic layering of polar–apolar groups, the charge alternation peak, and the van der Waals peak of nearest neighbors, respectively. For PILs, similar correlation distances are observed (denoted as I, II, and III), but with a significant difference: Peak I, representing backbone-to-backbone correlations, becomes the dominant feature, especially for poly­[BVIM]^+^[TFSI]^−^, revealing improved smectic layering.

**Figure 2:**
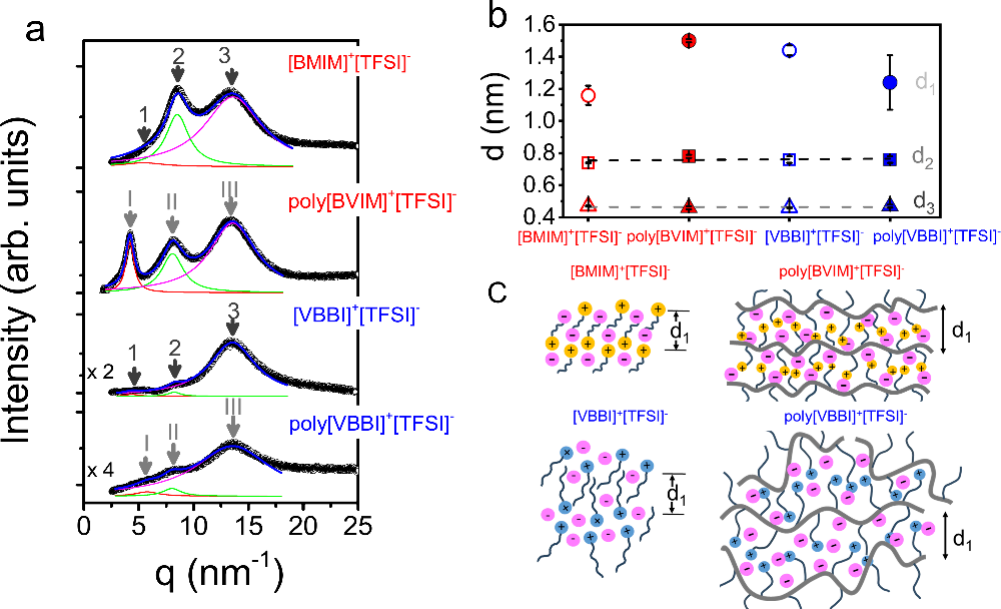
(a) XRD curves of the ILs and corresponding PILs at 358 K. For the ILs, arrows 1, 2, 3 indicate, respectively, the smectic layering of polar–apolar groups, the charge alteration peak, and the van der Waals peak of nearest neighbors. For the PILs, arrows I, II, III indicate, respectively, the distance between backbones, the anion–anion correlation distance, and the van der Waals contacts of atoms. Red, green and magenta lines are the respective component contributions to the scattering curve (blue). (b) Characteristic spacings (obtained as *d* = 2π/*q*) giving the period of smectic layering (*d*_1_), the charge alteration distances (*d*_2_) and the nearest approach of atoms (*d*_3_). The brown and gray dashed lines are linear fits to *d*_2_, *d*_3_. (c) Schematic representation of the correlation distances in the ILs and the corresponding PILs. The schematic depicts ordered backbones in the case of poly­[BVIM]^+^[TFSI]^−^, and relatively disordered domains in poly­[VBBI]^+^[TFSI]^−^.

Compared to [BMIM]^+^[TFSI]^−^ and poly­[BVIM]^+^[TFSI]^−^, correlations in [VBBI]^+^[TFSI]^−^ and poly­[VBBI]^+^[TFSI]^−^, appear weaker, suggesting a more disordered packing (Figure 2c). In poly­[VBBI]^+^[TFSI]^−^, the bulky phenyl-imidazole side group increases the rigidity of polymer backbone, resulting in an appreciable amount of packing frustration that disrupts the formation of a layered structure. In contrast, poly­[BVIM]^+^[TFSI]^−^ has a more compact structure with a smaller imidazolium-based side group, allowing for denser packing. This facilitates the formation of a more ordered layered domains. The variation in local packing can influence the anion transport. The impact on ionic mobility is reflected in the measured dc-conductivities for both IL and PILS pairs, as presented in Figure 3.

**Figure 3:**
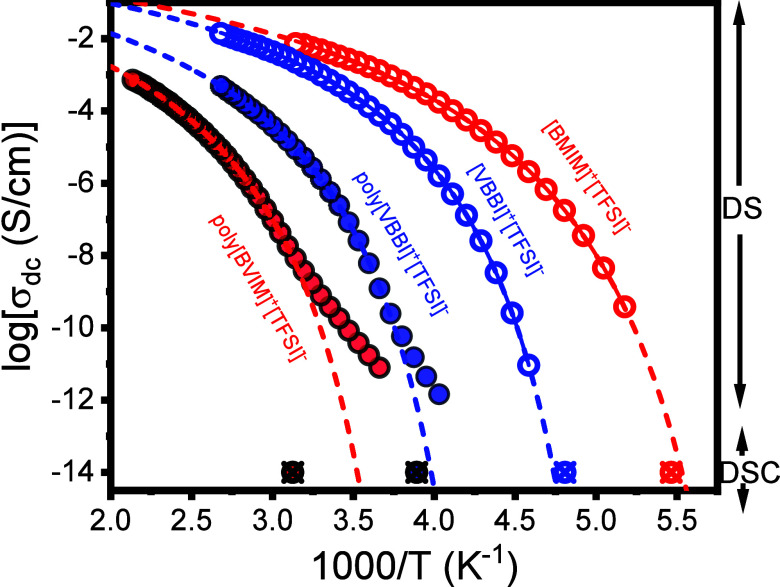
Temperature dependence of dc-conductivity for the ILs, shown with open circles, and corresponding PILs shown as spheres obtained on cooling; [BMIM]^+^[TFSI]^−^ (open red circles), [VBBI]^+^[TFSI]^−^ (open blue circles), poly­[BVIM]^+^[TFSI]^−^ (solid red circles), poly­[VBBI]^+^[TFSI]^−^ (solid blue circles). The solid/dashed lines are the results of fits to the Vogel–Fulcher–Tammann (VFT) equation. The crossed symbols represent the *T*_g_ obtained from DSC with a rate of 10 K·min^–1^.

The dc-conductivities and their dependence on temperature, σ′_dc_(*T*), shown in Figure 3. The values were obtained on cooling from the plateau at intermediate frequencies (Figure S3 **).** In general, σ′_dc_(*T*), follows the Vogel–Fulcher–Tammann (VFT) equation written for the conductivity contribution as

$$\sigma_{dc}\left(T\right)=\sigma_{0}\;\exp\left(-\frac{B}{T-T_{0}}\right)$$ 5

Here, σ_0_ is the dc-conductivity in the limit of very high temperatures, *B* is the activation parameter, and *T*_0_ is the “ideal” glass temperature (the parameters are summarized in Table 1, below). The glass temperatures of the ILs as obtained by DS are in good agreement with the DSC results. For poly­[BVIM]^+^[TFSI]^−^ and to a lesser degree for poly­[VBBI]^+^[TFSI]^−^, the σ_dc_(*T*) dependence follows the VFT dependence down to a temperature where a weaker (Arrhenius–like) dependence is obtained as

$$\sigma_{dc}\left(T\right)=\sigma_{0}^{\prime }\;\exp\left(-\frac{E}{RT}\right)$$ 6

Here *E* is the activation energy and R is the gas constant. At this temperature, ion dynamics decouple from the slower backbone dynamics as the system goes out of equilibrium. This temperature nicely corresponds to the DSC *T*_g_.

Figure 3 includes the estimated conductivity value at *T*_g_ as obtained from the Nernst–Einstein equation:

$$\sigma_{dc}=\frac{ne^{2}}{k_{B}T}\frac{{\left(\frac{d_{2}}{2}\right)}^{2}}{6\tau }$$ 7

Here, *e* is the elementary charge, $n=\frac{\rho N_{A}}{M}$ (ρ = 1.44 g·cm^–3^ applicable to [BMIM]^+^[TFSI]^−^) is the mass density, M = *M*_*w*_^cation^ + *M*_*w*_^anion^ is the molar mass of the cation and/or the backbone repeat unit and of the anion, *d*_2_/2 is the distance between two adjacent ions of opposite charge extracted from Figure 2, τ is the characteristic structural relaxation time at *T*_g_ (τ ∼ 100 s; the typical relaxation time associated with the dynamics at the liquid-to-glass temperature) and *k*_Β_ is the Boltzmann constant. Using *d*_2_/2 ∼ 0.38 nm in eq 7 results to σ_dc_ ∼ 10^–14^ S·cm^–1^. The obtained conductivity values were used to determine the DS glass temperature (Table 2). Due to the small size and low viscosity, ILs inherently exhibit higher dc conductivity as compared to their PILs counterparts. Conversely, within the PILs, poly­[VBBI]^+^[TFSI]^−^ demonstrates higher conductivity. This enhancement can be attributed to the electrostatic interactions between the [TFSI]^−^ anion and the phenyl-substituted imidazolium cation, taking place away from the backbone, that maintains significant mobility. In addition, the loose packing structure in poly­[VBBI]^+^[TFSI]^−^ prevent the formation of tightly bound ion clusters thus promoting segmental mobility. In contrast, in poly­[BVIM]^+^[TFSI]^−^, electrostatic interactions between the cation and anion occur in close proximity to the polymer backbone thus restricting backbone dynamics and limiting ion mobility. In support of this notion, the zero-shear viscosity of poly­[VBBI]^+^[TFSI]^−^ and poly­[BVIM]^+^[TFSI]^−^ was examined and plotted in Figure S4 as a function of the *T*_g_-scaled temperature. Across the entire temperature range, poly­[VBBI]^+^[TFSI]^−^ exhibits consistently lower zero-shear viscosity than poly­[BVIM]^+^[TFSI]^−^. These findings align with DSC results, as both conductivity and thermal behavior reflect the influence of the cation structure on ion dynamics. Similar effects could be observed in imidazolium based PILs of the type P­[ECnVim]^+^[PF_6_]^−^.^49^ There, increasing the size of the alkyl chain spacer *n* - therefore placing the ion association away form the backbone - resulted to an increased conductivity.

**Table 2:** Parameters of the VFT Equation for the Conductivity (Data Obtained on Cooling)

	*σ*_0_*/*(S·cm^–1^)	*B* (K)	*T*_0_ (K)	*T*_g_^DS^ (K) (at σ = 10^–14^ S/cm)	*T*_g_^DSC^ (K)^a^
[BMIM]+[TFSI]^−^	1.6	860 ± 10	155 ± 1	181 ± 1	183 ± 2
[VBBI]^+^[TFSI]^−^	1.5	880 ± 5	185 ± 1	210 ± 1	208 ± 2
poly[BVIM]^+^[TFSI]^−^	0.52	1505 ± 25	235 ± 1	284 ± 1	320 ± 2
poly[VBBI]^+^[TFSI]^−^	0.72	1150 ± 15	215 ± 1	252 ± 1	257 ± 2

a *T*_g_ obtained from DSC is also shown for comparison.

In conclusion, DS measurements of ion transport in the bulk ILs and PILs revealed that ion-conductivity in the latter is influenced by the cation size and in particular by the proximity of the cation to the backbone. In addition, anion dynamics where decoupled from the slower backbone on approaching *T*_g_ from higher temperatures and the decoupling was stronger for the backbone with the higher *T*_g_. In a second part we investigate the effects of confinement.

### Confinement and Flow Effects on Ion Transport

There exists a growing interest in the way ionic systems penetrate nanopores. ILs of [BMIM]^+^[Cl]^−^ where shown to penetrate AAO nanopores with their bulk viscosity.^48^ In bulk PIL the high zero-shear viscosity precluded any investigation of the imbibition kinetics. Instead, mixtures of PILs with their corresponding ILs had a lower zero-shear viscosity that allowed an investigation of the imbibition kinetics within AAO nanopores.^19^ It was shown that capillary action separates the IL from the PIL and effectively enriches the pores with the low viscosity component (IL).^19^ Herein, we carefully select the molar masses and monitor the imbibition kinetics of the two PILs by *ex situ* reflection optical microscopy (ROM), and by *in situ* nanodielectric microscopy (*n*DS). Figure 4a,c, gives the results of the imbibition length of poly­[VBBI]^+^[TFSI]^−^ and poly­[BVIM]^+^[TFSI]^−^ within AAO cross sections by ROM obtained for different time intervals. The imbibition length for the two PILs is plotted as a function of the square root of time in Figure 4b under conditions *ΔT* = *T*_imbibition_ – *T*_g_ = 100 K.

**Figure 4:**
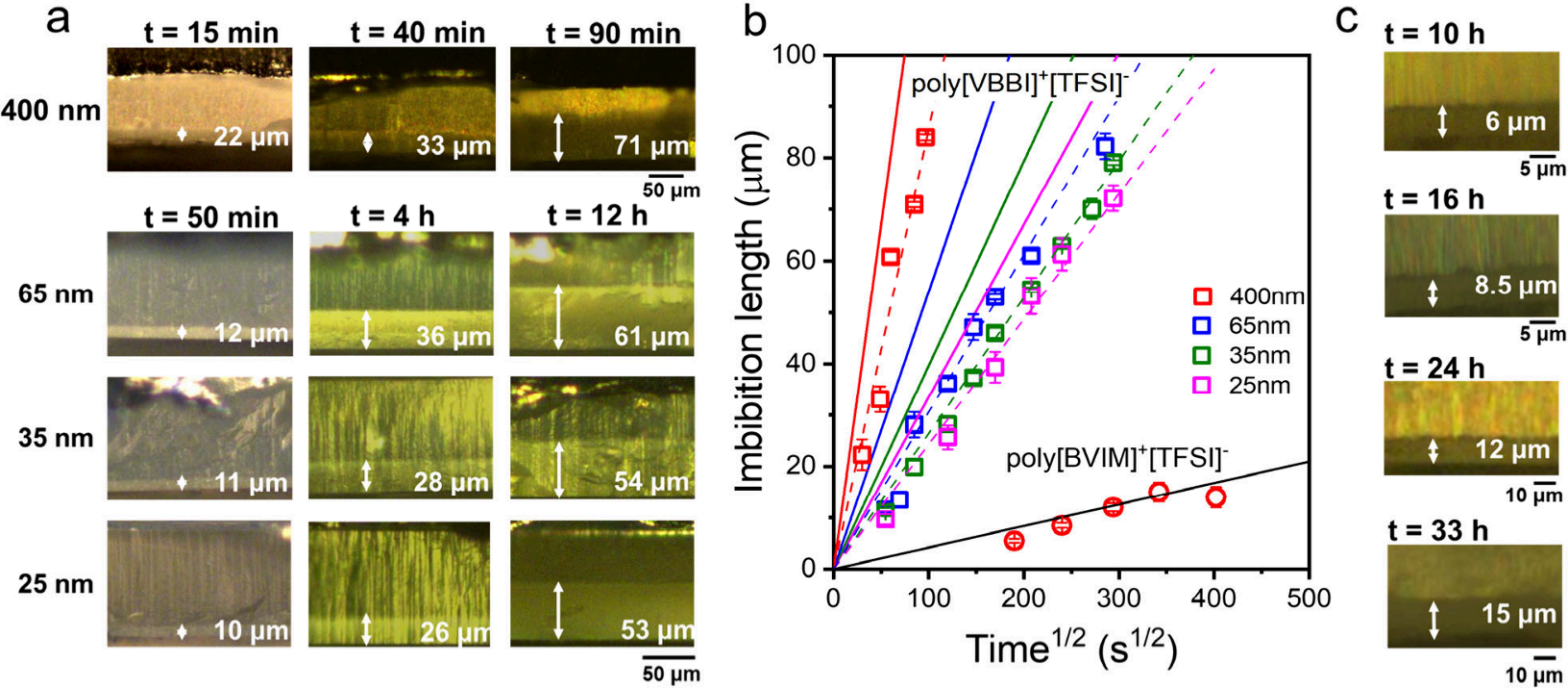
Optical images of the imbibition of (a) poly­[VBBI]^+^[TFSI]^−^ in AAO templates with different pore sizes measured at 358 K and (c) poly­[BVIM]^+^[TFSI]^−^ in AAO templates with a diameter of 400 nm at 400 K. At the corresponding imbibition temperature, the temperature difference from the respective *T*_g_ is constant (i.e.,*ΔT* = *T*_imbibition_ – *T*_g_ = 100 K for both PILs). The white double-headed arrows indicate the imbibition length at the specified time interval. (b) Imbibition lengths for poly­[VBBI]^+^[TFSI]^−^ and poly­[BVIM]^+^[TFSI]^−^ measured at 358 and 400 K, respectively, by *ex situ* ROM within AAOs with different pore sizes: 400 nm (red), 65 nm (blue), 35 nm (olive), and 25 nm (magenta). Solid lines are theoretical predictions based on the Lucas-Washburn equation (LWE) with parameters: η_0_ (bulk, poly­[VBBI]^+^[TFSI]^−^) = 10^3^ Pa·s, η_0_ (bulk, poly­[BVIM]^+^[TFSI]^−^) = 7 × 10^5^ Pa·s, γ_L_ = 27.4 mN/m, and cos θ = 0.5. Dashed lines represent the result of a linear fit to the data.

According to the Lucas-Washburn equation,^50,51^ the imbibition length, *L*, follows: $L={\left(\frac{\gamma R\;\cos\;\theta }{2\eta }\right)}^{1/2}\sqrt{t}$ , where γ is the surface tension, θ is the contact angle, η is the zero-shear viscosity, and *R* is the pore radius. The LWE was derived for Newtonian liquids. Figure 4a, c present ROM images with values of imbibition lengths obtained across several nanopores. Averaged *L* values are plotted in Figure 4b. These data together with the LWE can be used to extract an effective viscosity applicable to the PIL during flow in nanopores. For poly­[VBBI]^+^[TFSI]^−^, in all pore diameters investigated, the extracted effective viscosity (using cos θ = 0.5 and γ_L_ = 27.4 mN/m) is higher than in bulk (bulk η_0_ = 10^3^ Pa·s). The effective viscosities were determined as 1.9 × 10^3^, 2.4 × 10^3^, 1.7 × 10^3^ and 1.5 × 10^3^ Pa·s within pores of 400, 65, 35, and 25 nm, respectively. This deviation from bulk behavior is expected to influence the dc-conductivity values. For poly­[BVIM]^+^[TFSI]^−^, the imbibition length within 400 nm nanopores increases again with the square root of time, but imbibition is significantly slower compared to poly­[VBBI]^+^[TFSI]^−^. This suggests a higher effective viscosity (η = 1.1 × 10^6^ Pa·s) for poly­[BVIM]^+^[TFSI]^−^ that hinder infiltration despite the same Δ*T*_g_. The result is consistent with a higher zero-shear viscosity in the bulk (Figure S4).

We compare the imbibition kinetics in the two PILs by following the increase in conductivity at a selected frequency using *in situ n*DS. The comparison is made under the same temperature difference (Δ*T*) from the respective *T*_g_ in Figure 5. Poly­[VBBI]^+^[TFSI]^−^ demonstrates faster imbibition kinetics. Despite the identical Δ*T*, poly­[BVIM]^+^[TFSI]^−^ penetrate slower into the same nanopores. This is described by a higher effective viscosity for poly­[BVIM]^+^[TFSI]^−^ at the imbibition temperature. Indeed, the *T*_g_-scaled zero-shear viscosity (Figure S4) shows a 2 orders of magnitude higher zero-shear viscosity in poly­[BVIM]^+^[TFSI]^−^ as compared to poly­[VBBI]^+^[TFSI]^−^ at the imbibition temperature.

**Figure 5:**
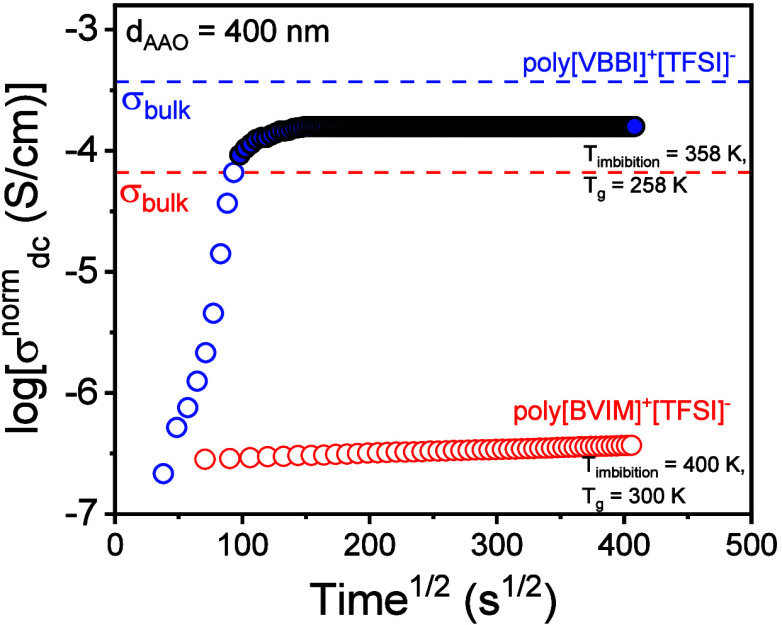
Comparison of the evolution in ionic conductivity from *in situ* nDS for poly­[VBBI]^+^[TFSI]^−^ (blue) and poly­[BVIM]^+^[TFSI]^−^ (red) within AAO with a pore size of 400 nm at different temperatures corresponding to the same temperature difference from the respective *T*_g_ (*ΔT* = *T*_imbibition_ – *T*_g_ = 100 K). The dashed lines respect the dc-conductivity of PILs in bulk.

Because of the extremely slow imbibition kinetics in poly­[BVIM]^+^[TFSI]^−^, we explored the pore-size dependence of the conductivity evolution in poly­[VBBI]^+^[TFSI]^−^. The results at the imbibition temperature of 358 K are shown in Figure 6. In 400 nm pores, there is a fast initial increase of ionic conductivity followed by a constant value (e.g., dc-conductivity) at later times (Figure 6a). The open symbols in Figure 5 and Figure 6b denote an initial rise of conductivity in the absence of a conductivity plateau, e.g., the values do not correspond to the dc-conductivity, but to the ac-conductivity (at a frequency of 10^5^ Hz). It is only at later times that the plateau is developed (Figure 6a). The time scale for the development of the conductivity plateau nicely corresponds with the complete filling of nanopores as suggested by ROM (Figure 4). We note that for all investigated pore sizes, the dc-conductivity following the full imbibition remains consistently lower than the bulk value. Based on our previous studies,^18,19^ this reduction can be attributed to polymer-pore wall interactions, which induced polymer adsorption and restricted the mobility of polymer segments. The adsorbed polymer chains inevitably immobilize some counterions at the pore walls. Since the latter are unable to diffuse, they do not participate in charge transport, leading to a reduced overall conductivity compared to the bulk.

**Figure 6:**
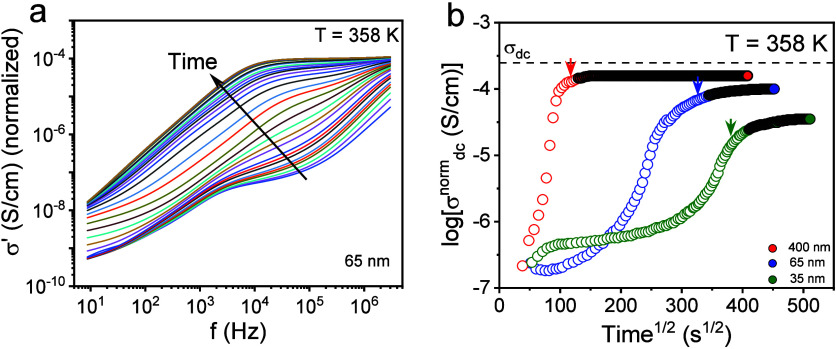
(a) Evolution of the real part of the normalized conductivity (for the porosity) of poly­[VBBI]^+^[TFSI]^−^ within AAO template with a diameter of 65 nm at 358 K. (b) Evolution of dc-conductivity during the imbibition of poly­[VBBI]^+^[TFSI]^−^ within AAO templates with varying pore sizes at 358 K. The conductivity values are corrected for porosity. The dashed line indicates the dc-conductivity for the bulk PIL at the same temperature. Open circles provide the (ac) conductivity of the poly­[VBBI]^+^[TFSI]^−^ during flow within AAO, while filled circles provide the respective dc-conductivities. Arrows indicate the time of full imbibition of the PIL, as determined from ex situ ROM experiments (Figure 4).

To explore confinement effects on the ion dynamics we performed temperature scans of the dc-conductivity starting from the imbibition temperature by cooling down to the glassy state. The dc-conductivity values can be discussed with the help of Figure 7. In addition, the figure includes the zero-shear viscosity in the bulk. In the same figure we plot the characteristic ion relaxation times as extracted from the modulus representation as M* = 1/ε* (Figure S3). At high temperatures, the dc-conductivity and the characteristic times of ion relaxation follows a VFT dependence, with the corresponding VFT parameters listed in Table 3. As we discussed earlier with respect to Figure 3, the σ_dc_(*T*) dependence for poly­[VBBI]^+^[TFSI]^−^ follows the VFT dependence down to a temperature where ion dynamics decouple from the backbone as the system goes out of equilibrium. As shown in Figure 7, the decoupling depends on pore diameter. At higher temperatures, the dc-conductivity is lower than the bulk because of adsorption during imbibition that drives some ions at the pore walls. At lower temperatures, confinement results in a stronger decoupling from the backbone dynamics according to eq 6. From an apparent activation energy (*E*) of 142 ± 6 kJ/mol in the bulk, to 109 ± 4 kJ/mol and to 108 ± 4 kJ/mol in nanopores with diameters of 400 and 35 nm, respectively. Spatial constraints alter local cation–anion correlations and modify the distribution of accessible pathways for ion transport.

**Figure 7:**
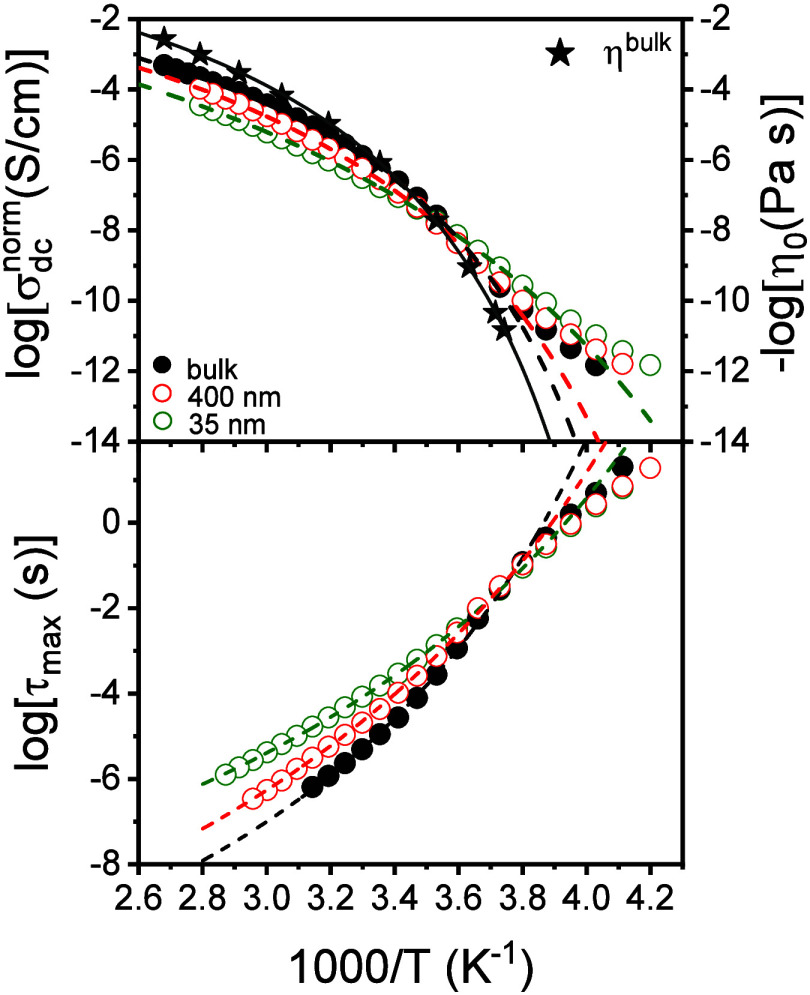
(Top) Temperature dependence of the dc-conductivity (left) and the zero-shear viscosity (right) for bulk poly­[VBBI]^+^[TFSI]^−^ (filled spheres) and confined poly­[VBBI]^+^[TFSI]^−^ in AAO nanopores with diameters 400 nm (red circles) and 35 nm (green symbols) following the imbibition experiments (shown in Figure 6b). (Bottom) Corresponding ion relaxation times extracted from the modulus representation plotted as a function of inverse temperature. In both cases the dashed lines represent VFT fits.

**Table 3:** Parameters of the VFT Equation for the poly­[VBBI]^+^[TFSI]^−^ pertinent to the Temperature Scan Experiments Following Imbibition

pore diameter, *d* (nm)	–log 10 (τ_0_/s)	*B*^τ^ (K)	*T*_0_^τ^ (K)	log_10_ [σ_0_/(S·cm^–1^]	*B*^σ^ (K)	*T*_0_^σ^ (K)
bulk	11 ± 0.1	1230 ± 30	215 ± 2	–0.14 ± 0.1	1150 ± 15	215 ± 1
400	12 ± 0.3	1970 ± 150	190 ± 5	0.19 ± 0.1	1500 ± 30	202 ± 1
35	12 ± 0.2	2650 ± 130	150 ± 4	0.44 ± 0.1	2060 ± 50	170 ± 2

				log_10_ [η_0_/(Pa·s)]	*A* (K)	*T*_0_ (K)
bulk	--	--	--	0.9 ± 0.1	1235 ± 40	220 ± 1

The activation energy in the bulk is in proximity to the predictions of a recent model discussing the dependence of the activation energy of ion diffusion on ion size.^30^ The activation energy for the ion diffusion shows a nonmonotonous dependence on the mobile ion size, indicating competition between Coulombic and elastic forces. It was suggested that the former dominates the mobility of small ions, while the latter controls mobility of large ions (e.g., TFSI). The term due to elastic forces has its origin on the shoving model of the glass “transition″.^52^ The model considers the motion of a structural unit in a frozen environment and suggests that the activation energy for molecular motion is controlled by thermal fluctuations creating an increase in local volume that is comparable to the volume of the moving ion. The reduced activation energy under confinement could suggest additional packing effects in the contribution of the elastic forces.

Overall PILs, contrary to ILs, penetrate AAO nanopores with a higher effective viscosity than the bulk (by approximately a factor of 2). Following imbibition, the conductivity data show the manifestation of adsorption and confinement affects. At higher temperatures, PILs exhibit reduced ionic conductivity as compared to bulk. Polymer adsorption controls ionic mobility in this limit. By decreasing temperature toward the bulk *T*_g_, anion motion decouples from the sluggish backbone dynamics and restores part of the ionic conductivity. Overall, confinement can be employed as a tool of decoupling anion dynamics from the backbone.

## Conclusion

Precise synthesis of ILs and of the corresponding PILs bearing identical anions but different cation sizes provided the means of testing the effects of ion association on ion mobility in the bulk and under nanometer confinement.

In the bulk PILs, ion conductivity was influenced by cation size, and in particular, by the distance of the cation from the backbone. Placing the cation near the backbone resulted to ion association that effectively rigidified the chain. This had an effect on the glass temperature (increasing) and on the ion conductivity (decreasing). In PILs there was a decoupling of ion motion from the backbone dynamics by decreasing temperature near *T*_g_; the higher the *T*_g_ of the PIL the stronger the decoupling. The temperature dependence of ion conductivity deviated from the Vogel–Fulcher–Tammann law and followed an Arrhenius temperature dependence with an activation energy of 142 kJ/mol in the case of poly­[BVIM]^+^[TFSI]^−^.

When the same PILs were located within nanopores, the ionic mobility was influenced by two effects: *adsorption* and *confinement*. PILs penetrated nanopores with higher effective viscosity than in bulk, reflecting adsorption effects. The latter effects dominated ion mobility at *T*≫*T*_g_ and gave rise to reduced conductivity. As temperature was lowered near *T*_g_, confinement enhanced the decoupling of ion motion from the polymer backbone by reducing the activation energy in the smaller pores. Under these conditions, the temperature dependence of ion conductivity followed an Arrhenius temperature dependence with an activation energy that was reduced to ∼ 108 kJ/mol under confinement. It suggested that confinement provides the means of increasing conductivity at temperatures in the vicinity of *T*_g_ where, intrinsically, ion mobility is reduced.

The results offer some new insights into how molecular structure and confinement affect ion transport in PILs. Placing the cation away from the polymer backbone and further confining the PIL with nanopores can increase the ionic conductivity at lower temperatures, and this, despite adsorption effects that act at higher temperatures but in an opposite direction.

## Supplementary Material



## Notes

Differential scanning calorimetry results for bulk ILs, dc-conductivity, and XRD results in smaller nanopores (PDF)
