# Supplementary material for: Tethered Cation Size Affects the Imbibition of Polymerized Ionic Liquids and the Ionic Conductivity in Nanopores
Source: Macromolecules. 2025 Jul 7;58(14):7534–43. doi: 10.1021/acs.macromol.5c01449 (PMC12288085; doi:10.1021/acs.macromol.5c01449)
Supplement: Supplementary file 1 [file ma5c01449_si_001.pdf]

Supporting Information

Tethered Cation Size Affects the Imbibition of Polymerized Ionic Liquids and the Ionic Conductivity in Nanopores

Yun Dong[a], Hongkun He[b], Kriti Kapil[b], Martin Steinhart[c]

Krzysztof Matyjaszewski[b], Hans-Jürgen Butt[a], and George Floudas[d,e,a] *

[a] *Max Planck Institute for Polymer Research, 55128 Mainz, Germany*

[b]*Department of Chemistry, Carnegie Mellon University, 15213 Pittsburgh, USA*

[c] *Institut für Chemie neuer Materialien, Universität Osnabrück, D-49069 Osnabrück, Germany*

[d] *Department of Physics, University of Ioannina, 45110 Ioannina, Greece*

[e] *University Research Center of Ioannina (URCI) - Institute of Materials Science and Computing, 45110 Ioannina, Greece*

**Corresponding author E-mail: gfloudas@uoi.gr*

ORCID George Floudas: 0000-0003-4629-3817

A. CHARACTERIZATION

Gel permeation chromatography (GPC). The molecular weight of poly[VBBI]$^+$[TFSI]$^-$ was measured using a series of PILs containing [TFSI]$^-$ anions synthesized by reversible addition-fragmentation chain-transfer (RAFT) polymerization from 4-vinylbenzyl chloride (denote as poly[VBBI]$^+$[TFSI]$^-_{RAFT}$) as the standards.[1,2] To prepare a solution of polymer for GPC measurement, *ca.* 2 mg of poly[VBBI]$^+$[TFSI]$^-$ was dissolved in 1 mL THF containing 10 mM LiTFSI and 10 mM 1-butylimidazole, and the solution was filtered through 0.2 μm polytetrafluoroethylene (PTFE) membrane filter before injecting into GPC columns. For poly[BVIM]+[TFSI]-, the linear PS standards was used for the calibration of the GPC measurement. The eluent was also THF solution containing 10 mM LiTFSI and 10 mM 1-butylimidazole.

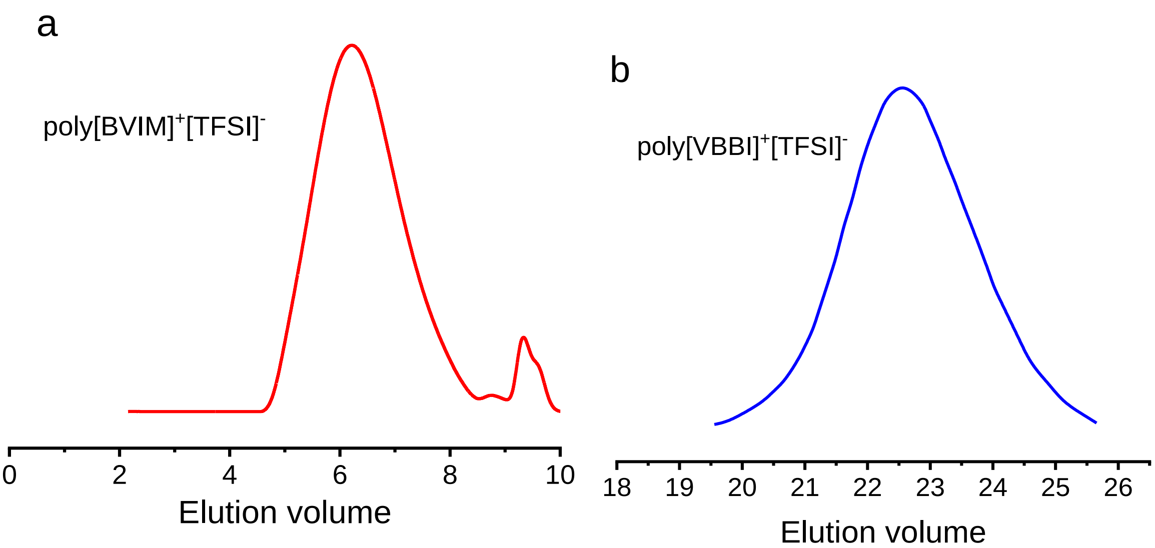

Figure S1. GPC curves of poly[BVIM]$^+$[TFSI]$^-$ (a)[3] and poly[VBBI]$^+$[TFSI]$^-$ (b)[1].

B. RHEOLOGY

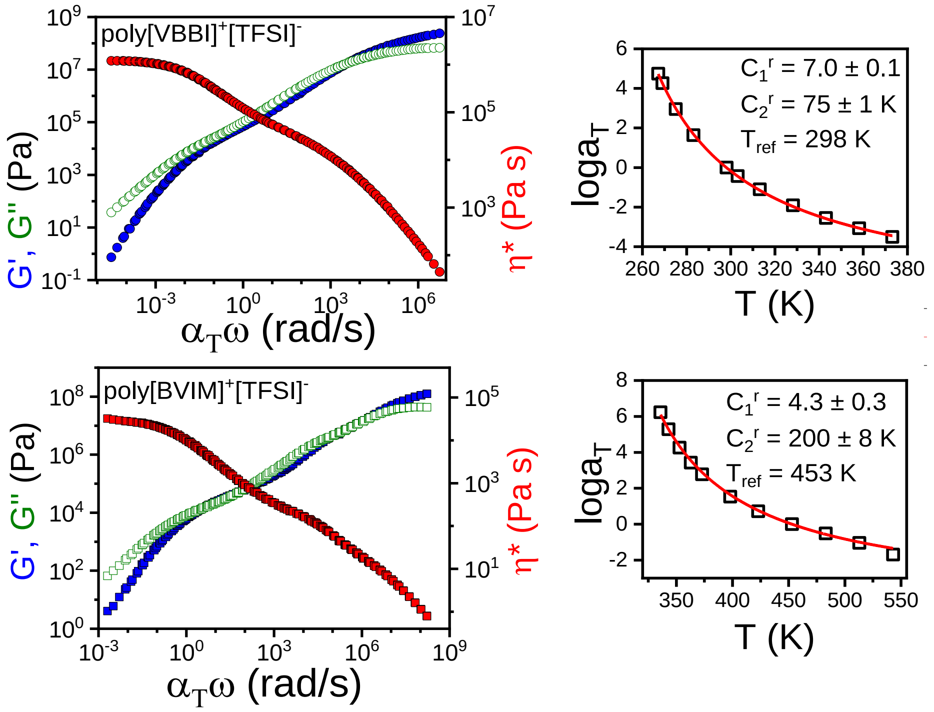

Figure S2. Master curves for the storage (blue), the loss (green) modulus and shear viscosities (red) of poly[VBBI]$^{+}$[TFSI]$^{-}$ and poly[BVIM]$^{+}$[TFSI]$^{-}$. Inset plots indicate the shaft factors at the corresponding reference temperature.

C. IONIC CONDUCTIVITY

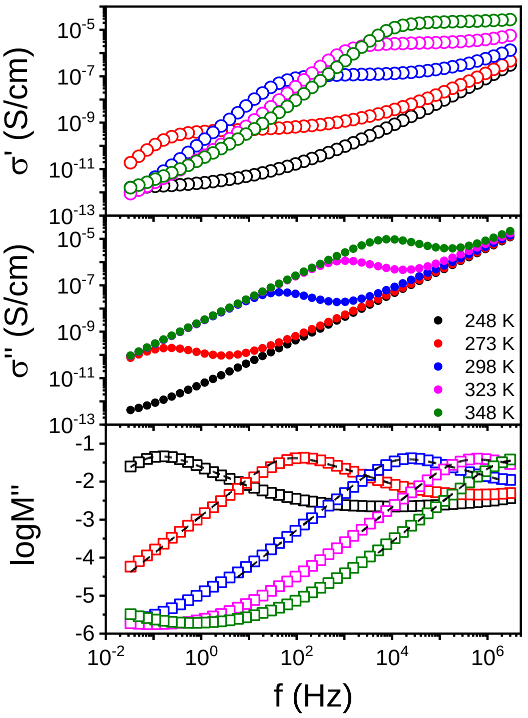

Figure S3. Real (top) and imaginary (middle) parts of the ionic conductivity of poly[VBBI]$^{+}$[TFSI]$^{-}$ in AAO templates with a diameter of 400 nm shown for the indicated temperatures. The modulus representation (bottom) is used to extract the characteristic relaxation times of ionic motion (dashed lines indicate representative fits).

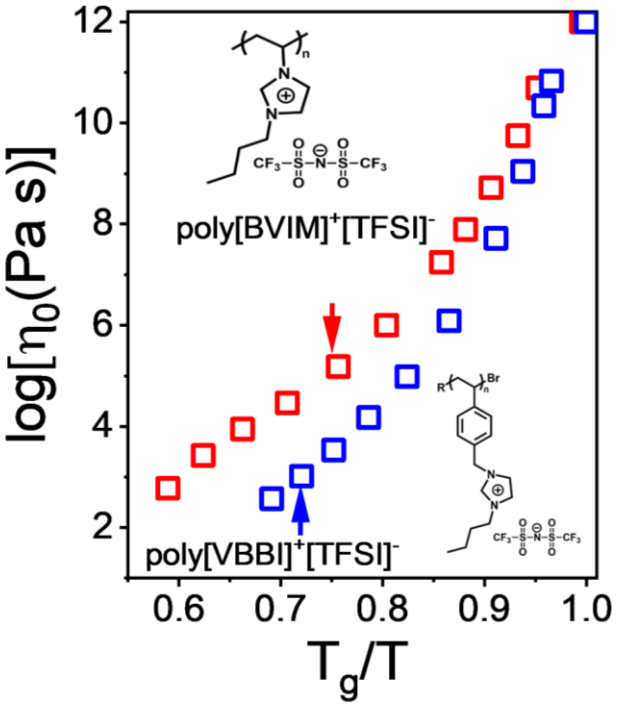

Figure S4. Temperature dependence of the T_g-scaled zero-shear viscosity (η_0) of poly[BVIM]$^+$[TFSI]$^-$ (red) and poly[VBBI]$^+$[TFSI]$^-$ (blue). Arrows indicate the imbibition temperatures (400 K and 358 K, respectively)

REFERENCES

[1] He, H.; Luebke, D.; Nulwala, H.; Matyjaszewski, K. *Macromolecules* **2014**, 47, 6601–6609.

[2] He, H.; Zhong, M.; Adzima, B.; Luebke, D.; Nulwala, H.; Matyjaszewski, K. *J. Am. Chem. Soc.* **2013**, 135, 4227−4230.

[3] Dong, Y.; Steinhart, M.; Butt, H. J.; Floudas, G. Demixing of Polymerized Ionic Liquid/Ionic Liquid Mixtures by Infiltration in Nanopores. *Macromolecules,* **2024**, *57,* 5409−5420.
